# Longitudinal associations between digital media use and ADHD symptoms in children and adolescents: a systematic literature review

**DOI:** 10.1007/s00787-022-02130-3

**Published:** 2022-12-23

**Authors:** Lisa B. Thorell, Jonas Burén, Johanna Ström Wiman, David Sandberg, Sissela B. Nutley

**Affiliations:** Department of Clinical Neuroscience, Karolinska Institutet, Stockholm, Sweden

**Keywords:** ADHD, Inattention, Hyperactivity, Impulsivity, Gaming, Social media, Longitudinal

## Abstract

Previous reviews have often shown a link between digital media ADHD symptom levels. However, longitudinal studies are needed to find stronger evidence of a causal effect as well as to determine the direction of effects. The aim of the present review (PROSPERO CRD42021262695) was therefore to provide a systematic review of studies meeting the following inclusion criteria: (1) include longitudinal data investigating associations between digital media (i.e., gaming and social media) and later ADHD symptoms or vice versa, (2) be published within the past 10 years (i.e., 2011 until June 2021), (3) be published in a peer-reviewed journal in English, and (4) include children or adolescents (age 0–17 years). After a systematic search in the Web of Science and PsycInfo databases, we included 28 studies, all with adequate or high quality. Results showed support for reciprocal associations between digital media and ADHD symptoms, with associations being more consistent for problematic use of digital media than for screen time. Thus, children with ADHD symptoms appear more vulnerable to developing high or problematic use of digital media (i.e., selection effects), and digital media also have effects on later ADHD symptom levels, either because of specific characteristics of digital media or because of indirect effects on, for example, sleep and social relations (i.e., media effects). However, it should be emphasized that further studies investigating potential moderators and mediators are needed if we are to better understand the complex associations between digital media and ADHD symptom levels.

## Introduction

Today, almost all teenagers in Western countries have their own smartphone [1], and the time spent using digital media (i.e., any activity that uses a digital device for leisure purposes such as playing digital games, using social media, or taking part of information or entertainment on the Internet) has increased radically during the past decade [2]. At the same time as children’s access to digital media has increased greatly, there has also been a large increase in the number of children diagnosed with Attention Deficit Hyperactivity Disorder (ADHD) [3]. This has led to the concern that digital media use might have effects on ADHD symptom levels. There are now several reviews and meta-analyses investigating the link between digital media in general and ADHD [4, 5], as well as more specific reviews focusing on the link between gaming and ADHD [6, 7] or ADHD symptom levels for individuals diagnosed with Internet gaming disorder [8, 9]. However, because this is an extremely fast-moving research area, there is a need for a new review that captures the current state of the field. In addition, none of the available reviews addressing this area has focused specifically on longitudinal relations. Longitudinal studies have the great advantage of being able to control for baseline levels and, thereby, to investigate to what extent digital media use influences changes in ADHD symptoms levels over time. It should also be noted that the association between digital media and mental health is most likely characterized by reciprocal relations in which both constructs affect each other over time [10], and longitudinal studies are able to investigate the direction of the effects. The overall aim of the present study was therefore to conduct a systematic review of longitudinal studies investigating the association between digital media and ADHD symptoms and vice versa.

### Digital media addiction

Previously, problematic media use has often been defined as entailing exposure to violent media content, whereas recent studies have focused more on the overarching negative consequences of digital media use. One important reason for this shift is most likely the introduction of “Gaming Disorder” (GD) in the 11th edition of the International Classification of Disease (ICD-11) [11] and “Internet Gaming Disorder” (IGD) as a diagnosis in need of further validation in the 5th edition of the Diagnostical and Statistical Manual of Mental Disorders (DSM-5) [12]. IGD includes nine different symptom criteria: (1) preoccupation with gaming, (2) withdrawal symptoms when gaming is taken away, (3) tolerance, (4) unsuccessful attempts to control gaming, (5) loss of interest in previous hobbies/activities as a result of gaming, (6) continued gaming despite psychosocial problems, (7) deception, (8) gaming to escape or relieve negative mood, and (9) jeopardizing relationships, job, or educational/career opportunities because of gaming.

Thus far, there has been no official recognition of addictive social media usage in DSM-5. However, it has been argued that criteria similar to those described above for IGD can also be applied to social media use [13–16]. It has also been suggested that a Social Media Disorder (SMD) should be included as a psychiatric disorder in the next version of the DSM, the argument being that social media use can be just as addictive and have just as serious effects on mental health as excessive gaming [16–19]. The current review is therefore not limited to gaming.

### Hypotheses linking digital media use and ADHD symptoms

Several hypotheses have been presented to explain how digital media use could be related to ADHD symptoms or vice versa. Some of these effects might apply to more general associations between digital media use and mental health problems, whereas some hypotheses focus on why digital media use might be associated with ADHD in particular. In addition, some of these hypotheses focus on explaining why digital media use can lead to increased levels of ADHD symptoms (i.e., media effects), whereas others focus on why children with ADHD symptoms might be more likely to develop problematic use of digital media later on (i.e., selection effects). It is important to emphasize that different hypotheses are not mutually exclusive—all of them could be correct to some degree, and they may also differentially explain the process on an individual level. It should also be noted that we can only speculate regarding this as randomized control trials (RCT), the best design for making causal inferences, is not possible to use within this area of research as few individuals are willing to refrain from using digital media. Experimental studies (e.g., ecological momentary assessment where the participant responds to a few questions several times per day) can provide much valuable information but are cumbersome to undertake as effects of digital media on ADHD symptom levels require relatively long study periods, and this design would therefore be overly intrusive in a person’s life.

One initial hypothesis is that the association between digital media use and ADHD symptoms represents a direct causal effect (i.e., something in the digital media content is directly causing symptoms of inattention, hyperactivity, and impulsivity). The “scan and shift hypothesis” [20] states that the fast pace of digital media may encourage using attentional resources to quickly scan and shift, making it more difficult to later engage in tasks requiring sustained attention. In addition, it has been hypothesized that children who have high levels of screen time have a harder time paying attention to less interesting activities, possibly because they lose the ability to regulate their attention internally after having gotten used to external regulation through digital media [21]. Violent media content has also been linked to ADHD symptoms, most likely because violent media are high in arousal, and the characters in this type of media often act impulsively [22]. It has also been shown that media multitasking is related to inattention, the hypothesis being that individuals engaged in media multitasking have difficulties focusing on one task, because they are accustomed to task-switching between media activities and other (offline or online) activities [23].

As described above, digital media use may have effects on ADHD symptoms, because such use leads to high arousal, which in turn leads to habituation and difficulties performing activities that are low in arousal. However, it should be noted that the direction of these effects is still unclear, as most studies have investigated cross-sectional correlations. According to the “Differential susceptibility to media effects model” [24], individuals are likely to select media content that fits within their existing dispositions. With regard to ADHD, it has been suggested that individuals with this disorder are more attracted than others to fast-paced activities, creating high arousal and delivering immediate rewards [5, 25]. Individuals with ADHD also often have problematic peer relations [e.g., 26] and show poor academic achievement [e.g., 27]. They may therefore use digital media as an escape from both reality and the negative feelings of being rejected, which in turn predicts the development of problematic digital media use as well as other mental health problems. Thus, escapism can be both a predictor of IGD and a mediator between digital media use and poor mental health [review by 28]. Of importance here is also the “social compensation hypothesis,” which states that individuals with poor social networks offline try to compensate for this by focusing more on online relations [e.g., 29]. In summary, children with ADHD symptoms may be more prone than other children to develop digital media addictions, with some individuals (e.g., those with poor social relations) being at particularly high risk. In addition, and as emphasized above, the links between ADHD and digital media use are likely reciprocal. This is sometimes referred to as the “Reinforcing Spiral Model” [10], which indicates that individuals with, for example, ADHD choose specific media content that is in line with their prevailing predisposition, which in turn may reinforce problematic behaviors.

In addition to direct causal effects of digital media use on ADHD symptom levels, it has been proposed that indirect causal effects could also be important. This hypothesis is sometimes referred to as the displacement hypothesis, as it suggests that time spent on digital media crowds out health-promoting behaviors, which in turn could increase ADHD symptom levels. Two of the most important activities commonly affected by extensive use of digital media are physical activity [e.g., reviews 30, 31] and sleep [e.g., 32]. In addition, excessive use of digital media early in life has been shown to be associated with lower levels of social interaction [e.g., 33] and delayed speech development [e.g., 34], which in turn can have a negative impact on peer relations. Thus, digital media use can affect several different health-promoting activities. These health-promoting activities are also linked to ADHD symptoms [35], and excessive use of digital media might therefore amplify pre-existing difficulties with maintaining unhealthy behaviors that children and adolescents with ADHD may already display due to the nature of their diagnosis. This could indicate that a small decrease in health-promoting behaviors has a larger negative impact on children with ADHD, because they start out at a lower level than their peers do. In the present review, we will therefore discuss to what extent the associations between ADHD and digital media use are affected by baseline levels (i.e., what level of problem behaviors or health-promoting behaviors the child already has), as well as to what extent effects of ADHD symptoms on later digital media use, or vice versa, are linear or non-linear.

Fourth, there is also a possibility that the observed association between digital media and ADHD symptoms is spurious and caused by a third variable, such as low socioeconomic status or male sex, factors known to be strongly associated with both ADHD and digital media use [4]. The present review will therefore provide an overview of the effects of different covariates.

### Results of previous reviews and meta-analyses

Although there are, thus far, no reviews of longitudinal studies examining the link between digital media use and ADHD symptoms, some previous reviews and meta-analyses including primarily concurrent data have provided valuable insights. Generally, the results of these studies show significant associations between ADHD symptoms and digital media use, but the strength of these relations varies greatly across studies depending on, for example, what type of sample has been studied and the type of digital media in focus. More specifically, several reviews [8, 9, 36, 37] have shown that there is an overrepresentation of ADHD among children/adolescents with problematic use of digital media (often defined as symptoms of IGD or Internet addiction). When investigating ADHD symptom levels rather than diagnosis, the results from previous reviews are less clear. Ferguson [6] concluded that computer gaming is not significantly associated with inattention, while another study [38] found an association between Internet use in general and ADHD symptom levels. Several previous reviews [4, 5, 22] have concluded that ADHD symptoms are significantly, but only weakly, associated with digital media use. However, this is most likely explained by the fact that many studies have controlled for different background variables (e.g., sex, age, SES, upbringing) in the statistical analyses rather than investigating their impact as moderators of the association of interest. This could suggest that there are subgroups of children with ADHD who are especially vulnerable to developing problematic use of digital media.

### Aim of the present review

As described above, there are both several plausible hypotheses and empirical support suggesting an association between digital media use and ADHD symptoms. However, the relatively large number of studies conducted recently are not included in these previous reviews, because they were published only a few years back. In addition, no previous review has focused on longitudinal studies. The overall aim of the present study was therefore to conduct a systematic review of longitudinal studies published within the past 10 years and examining the link between ADHD symptoms and digital media use. More specifically, the present study reviewed the previous literature with regard to the following research questions:

1. Is digital media use related to later ADHD symptom levels (i.e., media effects)?
2. Are ADHD symptom levels related to later digital media use (i.e., selection effects)?
3. In what way do covariates, mediators, and moderators influence the association between ADHD symptoms and digital media use and vice versa?

## Methods

### Search strategy

The updated Preferred Reporting Items for Systematic Reviews and Meta-analysis (PRISMA) [39] was used during the review process. A comprehensive literature search was conducted using PsycInfo and Web of Science. Reference lists of retrieved articles and review papers were also examined for any further studies. The exact search words used are presented in Table 1. Each of the studies found in the search was systematically and independently reviewed by at least two of the authors. In case of disagreement between the two raters, a third author was consulted, and a consensus decision was made between the three raters. The review was registered in PROSPERO (CRD42021262695).

**Table 1:** Search words

	Category	Search terms
1	Sample	Adolescen* OR teen* OR youth* OR child* OR girl* OR boy* OR kid*
2	ADHD	Neuropsychiatric OR adhd* OR”attention deficit*” OR”attention problem*” OR inattention OR hyperactiv*
3	Media	ALL = (gaming OR gamer OR gamification OR”computer game*” OR “video game*” OR “mobile game*” OR “Internet game*” OR “online game*” OR IGD OR “first person shooter” OR “strategy game*” OR “social media” OR “social network*” OR “digital media” OR “social platform*” OR “multiplayer game*” OR facebook OR snapchat OR Instagram OR tiktok OR twitter OR “Internet addiction” OR “media addiction” OR smartphone* OR likes OR”screen time” OR “media multitasking” OR “media use” OR “media exposure” OR “violent media” OR”screen-based”
4	Study design	longitudinal OR predict* OR follow-up OR prospective OR subsequent OR directionality
5	Year	2011 until present (June 2021)

### Inclusion and exclusion criteria

The inclusion criteria for papers to be reviewed were the following: (1) published within the past 10 years (i.e., 2011 until June 2021), (2) published in English, (3) published in a peer-reviewed journal, (4) including children or adolescents (age 0–17 years), and (5) using longitudinal data to investigate the relation between ADHD symptoms/diagnosis and digital media. Regarding the exclusion criteria, we did not include studies of children with ADHD recruited within a very limited setting (i.e., the criminal justice setting) or studies that only included children with ADHD and another comorbid medical or mental condition. In addition, we did not include the few available studies focusing exclusively on watching TV. The reason for this was that most children today do not watch digital media on a TV, but rather stream various types of digital media content (e.g., Netflix, YouTube) using several different platforms. For this reason, focusing on exclusively on TV watching does not capture the children’s digital media habits very well. We also excluded studies assessing ADHD symptoms using cognitive tasks (e.g., continuous performance tasks [CPT] to assess attention problems) as ratings and tests of inattention/impulsivity are not highly correlated and many tests of attention also measure other cognitive functions (e.g., speed of processing, working memory). Thus, including both ratings and tests would most likely have created too much heterogeneity.

### Included studies

**Fig. 1:**
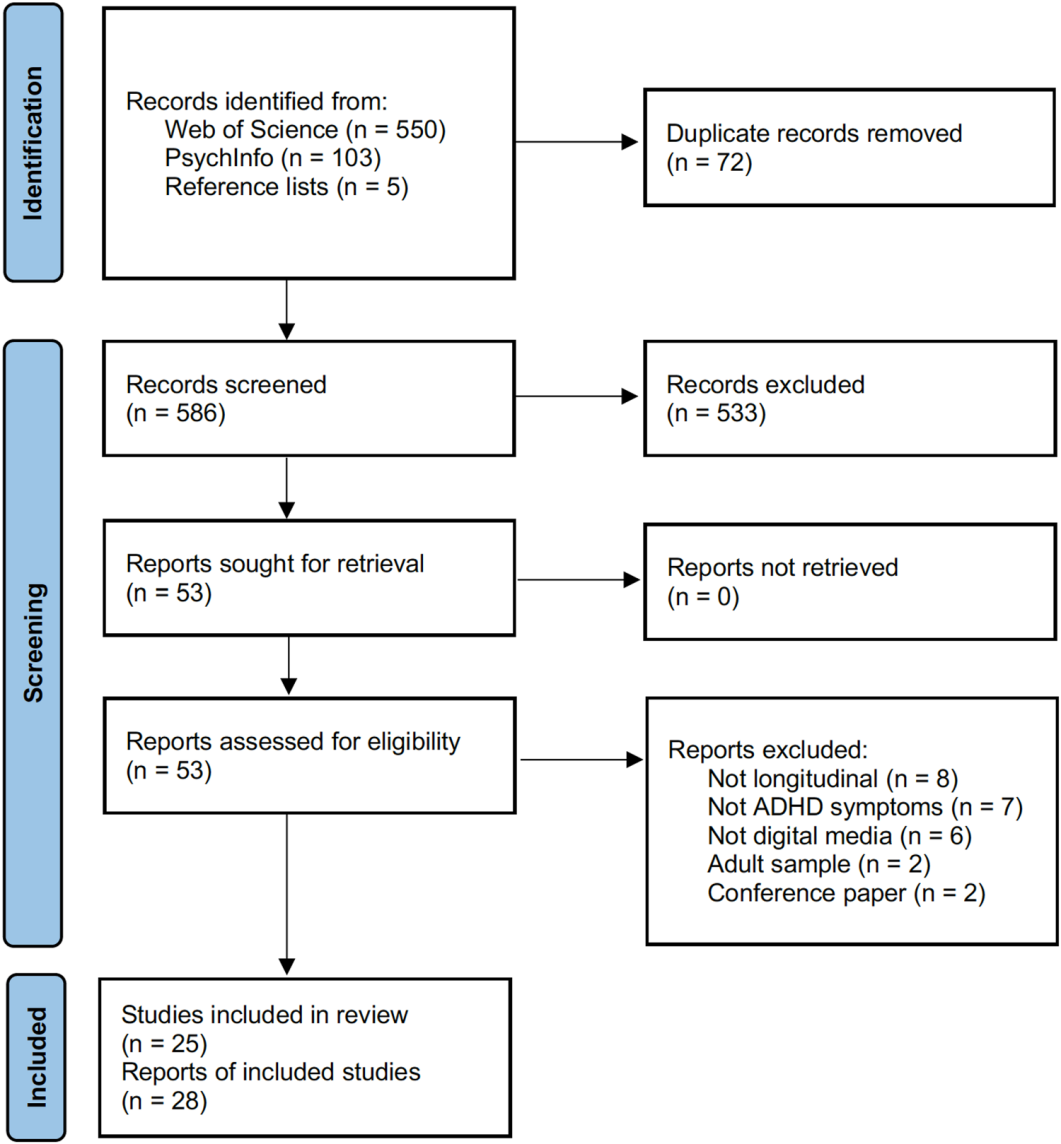
Flowchart

As shown in the flow chart presented in Fig. 1, a total of 586 references remained after removing duplicates. Of these, 533 reports were excluded based on the title and the information presented in the abstract. This left 53 reports for full-text screening. Out of these 53 reports, 25 were excluded, resulting in a total of 28 reports included in the review from altogether 25 different studies. The main reasons for exclusion of the report undergoing a full-text review were that the study (1) was not longitudinal (at least not for the analyses investigating relations between digital media and ADHD symptoms), (2) did not investigate ADHD symptoms specifically but rather a broader construct such as externalizing behavior problems, or (3) did not address digital media specifically but rather media use in general (e.g., reading books/magazines, listening to music).

The characteristics of the studies included in the review are presented in Table 2. Here, it is shown that the included studies varied in how they measured both ADHD symptoms and digital media. Most studies used some sort of ratings (self-reports, parent ratings, or teacher ratings), whereas three reports from the same project [40–42] used interviews to assess ADHD symptom levels and/or IGD symptoms. In addition, most studies (*n* = 19; 68%) used the same rater for both the predictor and the outcome measure, with 11 studies using only self-ratings and 8 studies using only parent ratings. For the studies using different raters at baseline and follow-up (*n* = 9), five studies used self-ratings to assess digital media use and parent ratings to assess ADHD symptom levels. The most used measures for assessing ADHD symptom levels (i.e., included in 23 out of 28 studies) were scales including the DSM-5 symptom criteria for ADHD (e.g., the ADHD rating Scale–IV) or the Strength and Difficulties Questionnaire (SDQ).

**Table 2:** Studies included in the systematic review (presented in alphabetical order), including quality ratings (QR)

Study (country) Sample size at baseline	Age at baseline (time span)	Measure digital media	Measure ADHD symptom levels	Control variables, mediator, and moderators	Direction of investigated effects	Results	QR
Allen and Vella 2015 [52] (Australia) *n* = 7818	6 and 10 years (2 years)	Screen time TV and digital games (P)	ADHD symptom levels (P)	Moderators: parental education and income, pubertal status, neighborhood SES, general health	DM → ADHD	Screen time was weakly (*β* = 0.04) but significantly associated with later ADHD symptom levels with control for screen time at baseline, but only for 10 years old (K cohort) and not for 6 years old (B cohort). Parental education and income, pubertal status, and neighborhood SES were not significant moderators in the longitudinal relations between screen time and ADHD symptoms levels	11
Barlett et al. 2012 [53] (USA) *n* = 1317	Grade 3–5 (6 and 13 months)	Screen time TV, video games and computer (S)	Inattention (T)	Control variables: Child sex, SES, parental education Mediator: sleep problems	DM → ADHD	Screen time at T1 (*r* = 0.19) and at T2 (*r* = 21) were related to inattention at T3 in the bivariate correlations. When including the other variables in the model, there was no direct effect of screen time on inattention. However, in the mediated model, screen time at T1 was related to sleep at T1 (*β* = − 0.26), which predicted sleep at T2 (*β* = 0.39) and then inattention at T3 (*β* = 0.05)	14
Baumgartner et al. 2018 [47] (Netherlands) *n* = 2390 (study 1) and 1083 (study 2)	11–16 years (3 and 6 months)	Screen time for digital multitasking (S)	Inattention (S)	Moderator: child sex and age group	DM → ADHD ADHD → DM	Digital multitasking was related to inattention both 3 and 6 months later with control for inattention at baseline, although only for younger adolescents (*b* = 0.16 and 0.19) in Study 1. Only significant effects for between-subject and not for within-subject analyses. Sex was not a significant moderator, but the correlations were twice as large for girls (*b* = 0.43) as for boys (*b* = 0.18) in Study 2. ADHD symptom levels were not significantly related to later multitasking	11
Beyens et al. 2020 [44] (Netherlands) *n* = 890	4–8 years (1 + 1 years)	Screen time for violent media and overall media use (P)	ADHD symptom levels (P)	Control variables: total screen time	DM → ADHD ADHD → DM	Screen time for violent media at T1 was not related to later ADHD symptoms. However, screen time for violent media at T2 was weakly related to ADHD symptoms at T3 (*r* = 0.08), but not in the crossed-lagged analyses (controlling for ADHD symptoms at baseline). Overall media use was related to later ADHD symptoms in all “between-subject” analyses (*β* = 20) but not in the “within-subject” analyses. ADHD symptoms at T1 (*β* = 17) and T2 (*β* = 0.15) were related to later screen time for violent media and this effect remained significant or marginally significant also in the “within-subject” analyses	11
Boer et al. 2020 [46] (Netherlands) *n* = 543	11–15 years (1 + 1 years)	Screen time and addiction of social media (S)	ADHD symptom levels (S)		DM → ADHD ADHD → DM	All bivariate correlations were significant. When controlling for ADHD symptoms at baseline, addiction to (*β* = 0.31–0.51), by not intensity (all *β* < 0.13) of social media was related to later inattention and to some extent impulsivity (but not hyperactivity). Effects were found for both “within-subject” and “between-subject” analyses. ADHD symptoms in relation to later digital media were not significant (all *β* < 0.26) when controlling for digital media at baseline	11
Chen et al. 2015 [63] (Taiwan) *n* = 1153	Grade 3, 5, and 8 (4 months)	Internet addiction (yes/no) screen time (including homework/e-mail) (S)	ADHD symptom levels (P)	Control variables: child sex, age, academic performance, school attitude/interaction, peer problems, parenting, social problems, family functioning, parenting, autistic traits, symptoms of ODD	ADHD → DM	Inattention (OR = 1.06) and hyperactivity/impulsivity (OR = 1.06) were weakly related to later Internet addiction but not to later screen time (both *β* = 0.04) when controlling for digital media use at baseline. However, academic performance and to some extent protective parenting and peer problems were the only significant predictors of addiction when including a range of different covariates	9
Ferguson and Ceranoglu 2014 [62] (USA) *n* = 144	Adolescents (M = 12.7 years) (1 year)	Symptoms of IGD and screen time (T1 = S; T2 = P)	Inattention (P)	Covariates: child sex and age, family attachment, peer criminality	DM → ADHD ADHD → DM	IGD symptoms (*β* = 0.05) were not related to later inattention when controlling for covariates (including ADHD symptoms at baseline). However, inattention (*β* = 0.19) was related to later IGD symptoms when controlling for IGD at baseline	9
Ferguson and Wang 2021 [60] (Singapore) *n* = 3034*	Youth (M = 11.2 years) (1 + 1 year)	Screen time violent games and computer games (S)	ADHD symptom levels (S)	Covariates: child sex and age, mother’s education, family environment, impulse control	DM → ADHD	No significant associations between screen time in general (*β* = 0.02) or screen time for violent games (*β* < 0.01) and later ADHD symptom levels when controlling for a range of different covariates (including ADHD symptoms at baseline)	9
Gentile et al. 2012* [54] (Singapore) *n* = 3034	8–17 years (1 year)	Screen time violent games and computer games (S)	ADHD symptoms (S)	Covariates: child sex, age, ethnicity, parental education and housing type (SES)	DM → ADHD ADHD → DM	In the time-lagged analyses, screen time for computer games in general (both *r* = 0.05), but not screen time for violent games (*r* = 0.03 and 0.00), was related to later symptoms of inattention and impulsivity, also when controlling for covariates and inattention at baseline. In the path analysis, a significant effect of screen time for gaming on later attention and impulsivity was found as well as a significant effect of inattention on later screen time for gaming	11
Hetherington et al. 2020 [48] (Canada) *n* = 1664	3 years (2 years)	Screen time for TV and computer games (P)	Inattention (P)	Moderator: parenting, group childcare	DM → ADHD	Screen time > 1 h/day at age 3 was associated with increased risk (OR = 1.69) of symptoms of inattention at age 5 years. Neither hostile/ineffective parenting, positive parenting, nor group childcare moderated this association. No control was made for inattention at baseline	9
Hirota et al. 2021 [68] (Japan) *n* = 5483	9–12 years (2 years)	Internet- addiction (S)	ADHD symptom levels (P)	Covariates: child sex, symptoms of autism, school grade (i.e., age)	ADHD → DM	Inattention (*β* = 0.05–0.08), but not hyperactivity (*β* = 0.00–0.03), was associated with both persisting pattern (i.e., stability of Internet addiction over time) and converting pattern (i.e., from no Internet addiction to Internet addiction) from T1 to T2, from T2 to T3 and from T1 to T3	14
Hygen et al. 2020** [42] (Norway) *n* = 703	10 years (2–4 years)	Symptom of IGD (interview S)	ADHD symptoms (interview S)		DM → ADHD ADHD → DM	Symptoms of IGD at age 10 (*r* = 0.10) were associated with ADHD symptoms at age 14 but not age 12 (*r* = 0.08). Symptoms of IGD at age 12 (*r* = 0.15) were associated with ADHD symptoms at age 14 (shown in supplementary material). When controlling for ADHD symptoms at baseline, symptoms of IGD were related to later ADHD symptoms in “between-subject” analyses but not in “within-subject” analyses. In the correlational analyses, ADHD symptoms at age 10 and 12 were related to later IGD symptoms at age 12 and 14 (*r* = 0.1–0.13) (supplementary material), but these effects did not remain significant in the path analyses	13
Jeong et al. 2020 [65] (South Korea) *n* = 2319	Grade 3, 4 och 7 (2 years)	Symptom of IGD (S)	ADHD symptom levels (P)	Covariates: child sex, age, family type, SES and child academic achievement. Mediators: self-esteem and aggression	ADHD → DM	ADHD symptoms at baseline were associated with symptoms of IGD at follow-up among both boys (*r* = 0.13) and girls (*r* = 0.17) in the bivariate correlations, but only for girls in the path model controlling for baseline levels. A mediating effect of low self-esteem (both sexes) and aggression (only for girls) was found in the path models. Small (17%) explained variance for the overall model including mediators	13
Liu et al. 2021 [49] (China) *n* = 2492	6 months and 2.5 years (1.5 and 3.5 years)	Screen time for TV and electronic devices: low/high (P)	ADHD symptom levels (P)	Covariates: child sex and age, parental education, siblings, passive smoking, outdoor activities, pregnancy factors (gestational age, birth weight, delivery mode)	DM → ADHD	Screen time (high versus low levels) at 6 months and at 2.5 years were related to ADHD symptom levels at age 4 years (both OR = 1.31), when controlling for a large range of covariates (although not ADHD symptoms at baseline). In separate analyses for boys and girls, screen time was only related to later ADHD symptom levels for boys	11
Männikkö et al. 2020 [51] (Finland) *N* = 8525	8 years (8 years)	Screen time for TV and computer games (S)	ADHD symptom levels (T1 = T; T2 = S)	Covariates: child sex and risk factors during birth and early life, emotional and behavioral variables (peer aggression, peer marginalization, behavior problems, fearfulness/ inhibition)	ADHD → DM	ADHD symptom levels at age 8 years were weakly associated with screen time 7–8 years later among both boys (*r* = 0.06) and girls (*r* = 0.12). Effects remained significant (*β* = 0.07) in the regression analyses when controlling for child sex, early risk factors, and several emotional and behavioral variables (but not screen time at baseline). Please note that follow-up data were collected already in 2000–2001	11
McNamee et al. 2021 [45] (the UK) *n* > 8000	10–15 years (1–6 years)	Screen time (high/medium/low levels) for social media (S)	ADHD symptom levels (S)	Covariates: child sex, age, ethnicity, maternal factors (education, mental health, employment), marital status, family income, region, year, urbanization, smoking, drinking, close friendships, number of siblings	DM → ADHD	Social media use > 4 h/day was associated with later hyperactivity and inattention (+ 0.85 points or over 20% of a standard deviation), also when controlling for ADHD symptoms at T1, as well as child and family factors and investigating individual fixed effects. Limited or moderate use of social media (less than 1 h or 1–3 h/day) was also associated with higher ADHD symptom levels, although the effect was smaller	11
Niiranen et al. 2020 [58] (Finland) *n* = 699	18 months (3,5 years)	Screen time (high/low) for programs and digital games (P)	ADHD symptom levels (P)	Covariates: child sex, age, maternal education, number of siblings and participation in full time day care	DM → ADHD	Screen time (low/high levels) at 18 months was not significantly related to either inattention or hyperactivity/impulsivity at 5 years of age, although odds ratios were relatively high (OR = 1.16–1.49). The average screen time at 18 months of age was low (mean = 34 min) and very few children (17 children; 2.8%) were therefore classified as having high levels of screen time	7
Parkes et al. 2013 [55] (the UK) *n* = 11,014	5 years (2 years)	Screen time for TV/video and computer games (P)	ADHD symptom levels (P)	Covariates: child sex, age, SES, maternal factors (education, income, physical and mental health, ethnicity, family composi-tion), family factors (mother–child relationship, parent–child joint activities, household chaos) and child factors (sleep problems, intelligence, physical activity, negative school attitude)	DM → ADHD	Both screen time for TV/video (*β* = 0.22) and digital gaming (*β* = 0.32) for more than 3 h/day, but not screen time for 1–3 h (*β* = 0.05 and 0.03), were associated with higher ADHD symptom levels compared to not gaming at all. Effects remained when controlling for baseline sex, age, and ADHD symptoms, but not in the final model when controlling for a range of child, mother, and family factors. Maternal and family characteristics produced the greatest reduction in the effect of screen exposure	10
Peeters et al. 2018 [66] (Netherlands) *n* = 544	11–15 years (1 year)	Symptoms of IGD (S)	Inattention (S)	Covariate: child sex Moderators: close friendships, life satisfaction	ADHD → DM	Inattention was related to later IGD symptoms when controlling for child sex and IGD symptoms at baseline (*β* = 0.30). The effect of inattention on later symptoms of IGD was stronger for adolescents who had fewer close relationships and who reported lower life satisfaction (i.e., significant moderators)	9
Poulain et al. 2018 [56] (Germany) *n* = 527	2–6 years (1 year)	Screen time TV/video, phone or Internet/computer (normal/high use) (P)	ADHD symptom levels (P)	Moderators: Child sex and age, SES	DM → ADHD ADHD → DM	Mobile phone use (OR = 1.10), but not computer/Internet or TV/video use, was associated with later ADHD symptoms when controlling for sex, age, SES, and ADHD symptoms at baseline. High use of mobile phones was also associated with a much-increased risk (OR = 3.36) of being classified as belonging to the ADHD symptom group. However, ADHD symptoms at T1 were not associated with any type of digital media use at follow-up. High use of mobile phones. Child sex, age, and SES were not significant moderators	7
Poulain et al. 2019 [59] (Germany) *n* = 814	10–17 years (1 year)	Screen time TV, phone or Internet/computer (S)	ADHD symptom levels (unclear rater)	Covariates: child sex, age, SES	DM → ADHD ADHD → DM	No type of screen time (phone, computer/Internet or TV/video) was associated with ADHD symptoms 1 year later when controlling for ADHD symptoms at baseline (*β* = − 0.01 to 0.03). ADHD symptoms were not significantly associated with later screen time (normal/high) when controlling for screen time at baseline	9
Ra et al. 2018 [57] (USA) *n* = 2645	15–16 years (21–23 months)	Frequency of using 14 digital media activities (S)	ADHD symptom levels (S)	Covariates: child sex, age, subsidized lunch eligibility, ethnicity, delinquency, depression, child and family substance use	DM → ADHD	High-frequency engagement in each additional digital media activity at baseline was associated with significantly higher odds (OR = 1.11) of having symptoms of ADHD across follow-ups. Effects remained significant when controlling for a range of different covariates (including ADHD symptom levels at T1). Adolescents above a threshold for ADHD symptoms at baseline were excluded from the study	11
Rydell and Brocki 2021 [50] (Sweden) *n* = 88	15 years (1 year)	Screen time violent media (S)	ADHD symptom level (S, P)	Covariates: CU traits, ODD symptoms Moderator: child sex	ADHD → DM	Symptoms of inattention (*r* = 0.28), but not symptoms of hyperactivity/impulsivity (*r* = 0.15), predicted violent media use 1 year later, although only for boys. Effects did not remain significant when controlling for callous unemotional (CU) traits. No analyses were made controlling for violent media use at T1	10
Stenseng et al. 2020** [40] (Norway) *n* = 791	7 years (2 + 2 years)	Screen time for computer games (P + interview S)	ADHD symptom levels (interview F)	Covariates: child sex, SES and emotional symptoms (depression and anxiety) Moderator: low/high gaming	DM → ADHD ADHD → DM	Some weak associations between screen time and later ADHD symptom levels without control for baseline, but these relations did not remain significant when controlling for screen time at baseline. ADHD symptom levels at age 6 were not associated with screen time at age 8 (*β* = 0.06), but ADHD symptom levels at age 8 (*β* = 0.16) were related to screen time at age 10 when controlling for sex, SES, emotional symptoms, and gaming at age 8). Associations were not significantly different between those who played a lot (“dedicated gamers”) and those who played a little (“causal gamers”)	12
Wartberg et al. 2018*** [61] (Germany) *n* = 1095	12–14 years (1 year)	Symptoms of IGD (S)	ADHD symptom levels (S)	Covariates: child sex, SES, antisocial behaviors, aggression, emotional distress, self-esteem, parental anxiety and depression	DM → ADHD ADHD → DM	IGD symptoms at T1 were related to ADHD symptom levels at T2 (*r* = 0.31), but results were mixed when controlling for the overlap between different predictors (including IGD symptoms at baseline), with an effect of IGD (*β* = 0.06 and 0.07) only being found in the model with restrictions (i.e., equal coefficients for boys and girls). ADHD symptoms at T1 were associated with IGD symptoms at T2 (*r* = 0.38) in a sample where 70% were classified as high risk of IGD at T1. Effects of ADHD remained significant (*β*s = 0.13 to 0.14) also when controlling for the overlap between different predictors (including baseline levels	11
Wartberg et al. 2021*** [67] (Germany) *n* = 1095	12–14 years (1 year)	Symptom of IGD and Internet addiction (S)	ADHD symptom levels (P)	Covariates: child sex, SES, antisocial behaviors, aggression, emotional distress, self-esteem, parental anxiety and depression	ADHD → DM	In the multiple regression analyses (i.e., controlling for the overlap between different predictors), ADHD symptoms at T1 were associated with symptoms of IGD (OR = 1.17), but not with problematic Internet use (OR = 1.08)	11
Wichstrøm, et al. 2019** [41] (Norway) *n* = 740	8 years (2 years)	Symptoms of IGD (interview S)	ADHD symptom levels (interview S)	Covariates: demographic variables (sex, ethnicity, income and education), family climate, self-esteem, temperament, emotion regulation, intelligence, executive deficits, bullying, physical activity, social skills, participation in sports, ODD, anxiety disorders, depression	ADHD → DM	ADHD symptom levels at age 8 years were weakly associated with symptoms of IGD at age 10 years in the bivariate correlations, but not when controlling for screen time at baseline (*β* = 0.04). When controlling for gaming at age 8 and child sex, the following predictors were significant: parental education, social competence, emotion regulation, intelligence, and involvement in organized sports. Results were similar for heavy involvement in gaming and negative consequences of gaming	11
Yang et al. 2014 [64] (South Korea) *n* = 1173	13–14 years (2 years)	Screen time for computer games, cyber bullying and online sexual exposure (S)	ADHD symptom levels (P)	Covariates: demographic variables (sex, height, weight, school performance, pocket money, birth order, SES, and family structure). Covariates: depression, anxiety, self-esteem, coping, internalizing problems, conduct problems, social relations, traditional bullying	ADHD → DM	ADHD symptom levels were associated with online bullying (OR = 1.61), but not online sexual exposure (OR = 1.25) or high screen time (> 3 h/day) for computer games (OR = 1.14). However, this effect did not remain significant in the multiple regression analyses when controlling for the overlap between ADHD symptom levels, demographic variables, and the additional predictors (including bullying experiences at baseline)	10

*DM* digital media, *IGD* internet gaming disorder, *P* parent rating, *S* self-rating, *SES* socioeconomic status, *T1* time point 1 1, *T2* time point 2, *T3* time point 3, *T* teacher rating

*These studies include the same sample; **These studies include the same sample; ***These studies include the same sample

### Quality ratings

Articles were assessed for quality utilizing the Quality Assessment Tool for Observational and Cohort and Cross-Sectional Studies created by the National Institutes of Health [43]. This scale includes 14 criteria and items related to internal validity, external validity, and study power. Each criterion was rated as 0 (i.e., criterion not met) and 1 (criterion met). Thus, the possible range was 0–14. Regarding Criterion 5 (i.e., power/sample size), an adequate power calculation or a sample size above 1000 participants at baseline was required for this criterion to be met. For Criterion 7 (i.e., sufficient time frame), we set the limit at 1 year for at least one of the follow-ups. Concerning Criterion 12 (i.e., blinding of outcomes assessors), we regarded this criterion as having been met if the outcome was assessed using an objective measure (i.e., interview) or if the study included separate raters for the predictor and outcome variable. For Criterion 13 (i.e., retention rate), we followed the recommendations and set the limit to at least 80%. A score of 0–6 was considered poor, 7–10 adequate, and 11–14 high quality. Two of the authors (LBT and JB) performed the quality ratings independently, and when necessary, reached a decision by consensus. However, it was only for criterion 13 (i.e., retention rate) that some inconsistencies between raters were found as this information was sometimes not very clearly described in the articles.

## Results

### Description of the included articles

As shown in Table 2, as many as 14 (50%) of the studies included in the review were published during 2020 or the first half of 2021, even though the time span for inclusion was the past 10 years. Many of the studies included large sample sizes (i.e., 17 studies with a sample size > 1000 at baseline), and the total number of participants at baseline in the studies was just above 66,000 taking into consideration that some of the studies were from the same project and therefore included the same individuals (see footnote in Table 2). Regarding the participants’ age, the studies included preschool children (*n* = 5), children aged 6–12 years (*n* = 9), or adolescents (*n* = 11), but a few studies included children from a larger age range (*n* = 3). The time span from baseline to follow-up ranged from 3 months to 8 years, with most studies using a follow-up period of about 1–2 years. It should be noted that all included studies examined symptom levels of inattention, impulsivity, and hyperactivity on a dimensional level (i.e., from low to high symptom levels) rather than focusing on ADHD diagnosis. All studies included between-subject effects and five studies [42, 44–47] also investigated within-subject effects. Most studies reported between-subject effects both with and without control for baseline levels (i.e., controlled for digital media use at baseline when examining associations between ADHD symptoms and later digital media use, or vice versa). However, four studies [48–51] failed to control for baseline levels altogether.

Regarding digital media use, the measures used in the studies included here can be classified into two broad categories: studies investigating screen time (i.e., time spent using some type of digital media) and studies investigating digital media addiction (i.e., excessive use and negative consequences of using digital media). Regarding screen time, the studies can also be classified into the following categories, with some studies including more than one type of digital media: digital media in general (*n* = 8), violent media (*n* = 3), gaming (*n* = 9), social media (*n* = 2), multitasking (*n* = 1), and cyberbullying/sexual exposure (*n* = 1). Regarding addiction, the included studies investigated Internet addiction in general (*n* = 3), gaming (*n* = 7), or social media (*n* = 1). An equal number of studies (19 out of 28) investigated digital media in relation to later ADHD symptom levels and the reverse relation. Thus, 10 of the included studies included reciprocal relations between digital media and ADHD symptoms. A brief description of how digital media and ADHD symptoms were assessed can be found in Table 2 (i.e., columns 2 and 3). In case a distinction was made between the different sub-symptoms of ADHD, this is clarified in the description of the results in column 6.

The quality ratings (QR) for the included studies are presented in the last column of Table 2. The total mean score on the quality assessment tool was 10.57, with scores ranging from 7 to 14. None of the included studies was considered to be of poor quality; 11 studies (39%) were of adequate quality and 17 studies (61%) were of high quality. Almost all studies met Criterion 1 (i.e., objectives and hypothesis) and Criterion 2 (i.e., study group description). In addition, all studies used reliable and valid measures of ADHD symptom levels, whereas only 16 studies (57%) provided information about the reliability of the measure used to assess digital media. Concerning Criterion 13 (i.e., retention rate), 16 studies (57%) had a retention rate of at least 80%. The lowest percentages were found for Criterion 10 (i.e., repeated exposures) with only 32% of the studies including more than two time-points and Criterion 11 (i.e., blinding), with only 36% of the studies using objective measures (i.e., interview) or different raters for the predictor and the outcome measures.

### Overall findings

**Fig. 2:**
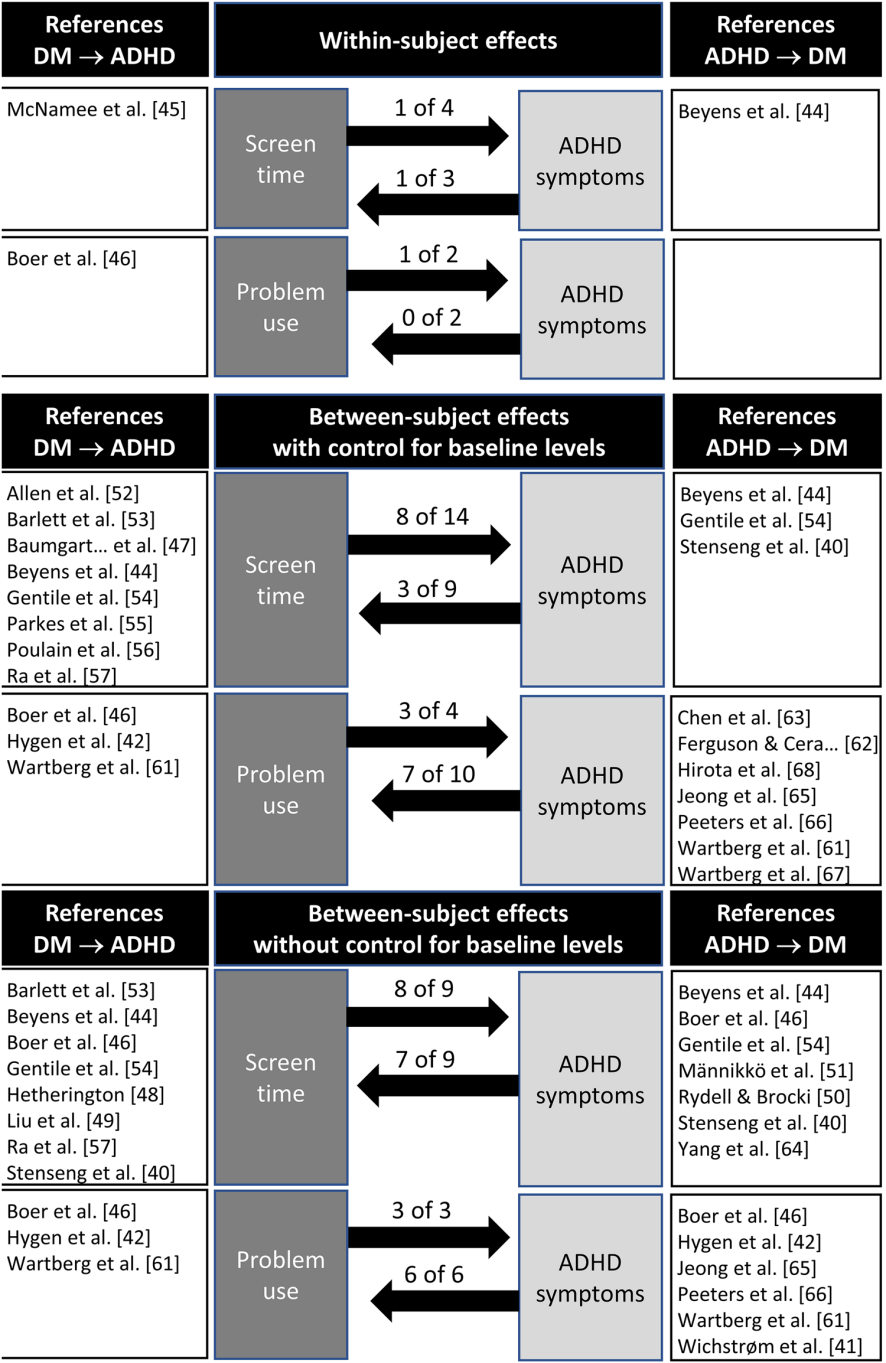
Summary of the results displaying the number of studies showing significant associations between ADHD symptom levels and digital media (DM)

As shown in Table 2, many of the studies included in the present review found significant associations both of digital media use on later ADHD symptoms and of ADHD symptoms on later digital media use. However, it is important to note that sample sizes were often very large, which means that the power to detect even small effects was high. In general, the size of the effects was relatively small (see last column of Table 2). As the included studies used many different types of analyses and a range of different covariates, a comparison between studies is difficult. However, results indicated that associations between problematic use of digital media and ADHD symptoms were somewhat more common and were stronger than associations between screen time and ADHD symptoms. In the case of significant associations, they were always positive (i.e., high levels of digital media being associated with high ADHD symptom levels or vice versa). To provide an overview of the results, a summary is presented in Fig. 2. This figure shows that significant associations in both directions (i.e., digital media in relation to later ADHD symptom levels and vice versa) were almost always found when not controlling for baseline levels. A substantial proportion of the studies (i.e., 33–75%) also found significant associations in between-subject analyses when controlling for baseline levels, whereas within-subject analyses were uncommon and only a minority of available studies found significant associations. These findings are presented in more detail below.

### Digital media in relation to later ADHD symptom levels

#### Screen time

Regarding studies investigating screen time in general (i.e., often an aggregated measure of use of computers, game consoles, mobile phones, and TV), the results of the current review show that eight studies found significant positive associations with later ADHD symptom levels or with symptoms of inattention when controlling for baseline levels of screen time [44, 47, 52–57]. Beyens et al. [44] only found associations between overall media exposure and later ADHD symptoms in between-subject analyses and not in within-subject analyses. In addition, three studies [40, 48, 49] only found relations when not controlling for baseline levels. The review also includes two studies that failed to find any significant associations between screen time in general and later ADHD symptom levels. Of these studies, Niiranen et al. [58] investigated screen time in children as young as 18 months in relation to ADHD symptom levels at age 5, where the cutoff for excessive screen time was defined as more than 45 min/day. Paulain et al. [59], who included a sample of children aged 10–17 years, found significant associations between use of computers/Internet and several other outcomes (e.g., peer relations, well-being, and quality of life), but not for ADHD symptom levels.

In studies investigating screen time for violent media (usually violent computer games) and later ADHD symptom levels, one study failed to find a significant association [60], while another study [54] found a significant association, although it did not remain significant when controlling for ADHD symptom levels at baseline. A third study [44] found that screen time for violent media at Time 1 was not related to ADHD symptoms at Time 2. However, screen time for violent media at Time 2 was significantly related to ADHD symptom levels at Time 3, although not when controlling for earlier ADHD symptom levels.

With regard to more specific measures of screen time, McNamee et al. [45] found a significant relation between screen time for social media and later ADHD symptom levels in within-subject analyses, whereas Boer et al. [46] only found a significant association when not controlling for baseline levels of social media use. Finally, one study [47] found a significant association between screen time for media multitasking (i.e., using several different media simultaneously) and later ADHD symptom levels in between-subject but not in within-subject analyses.

#### Digital media addiction

Although studies investigating the association between digital media addiction and ADHD symptoms have grown in number during the past few years, only four of them were longitudinal. Hygen et al. [42] found that symptoms of IGD at age 10 years were related to ADHD symptom levels both 2 and 4 years later, although these associations were only found in the between-subject (with and without control for baseline levels) and not in the within-subject analyses. Wartberg et al. [61] also found a small association between symptoms of IGD and later ADHD symptom levels when controlling for baseline levels of IGD symptoms, whereas Ferguson & Ceranoglu [62] failed to find such a relation. Here, it should be noted that the latter study included a few participants (*n* = 144), the majority of whom did not display a single symptom of IGD. Finally, Boer et al. [46] found a significant association between problematic social media use and later symptoms of inattention and to some extent impulsivity, but not hyperactivity. Associations were found for both between-subject (with and without control for baseline levels) and within-subject analyses.

### ADHD symptoms in relation to later digital media use

#### Screen time

Regarding ADHD symptom levels in relation to later screen time, seven studies found at least some significant associations, but only three studies found associations when controlling for ADHD symptom levels at baseline. Of the studies finding significant associations with control for baseline, Beyens et al. [44] found associations to screen time for violent media in both between-subject and within-subject analyses. In addition, Stenseng et al. [40] found that ADHD symptoms at age 6 were related to time spent on computer gaming at age 8. However, ADHD symptoms at age 8 were not related to time spent on computer gaming at age 10. Finally, Gentile et al. [54] found that attention problems were related to later video game playing. The studies that failed to find associations when controlling for ADHD symptom levels at baseline (or failed to control for baseline levels altogether) investigated screen time in general [51, 63, 64], screen time for multitasking [47], social media [46], violent games [50], and Internet bullying/sexual harassment [64].

#### Digital media addiction

Regarding studies examining associations between ADHD symptoms and later gaming addiction, all studies except three [41, 42, 46] found significant relations [61–63, 65–67], when controlling for baseline levels. In addition, Hirota et al. [68] found that inattention, but not hyperactivity, was associated with both the persisting pattern (i.e., stability of Internet addiction across time) and the converting pattern (i.e., from no Internet addiction to Internet addiction) across 2 years. Interestingly, two studies [41, 42] not finding significant associations when controlling for baseline levels used interviews to assess gaming addiction. The only study [46] investigating ADHD symptoms in relation to later social media addiction only found significant associations when not controlling for baseline levels of ADHD symptoms. It should also be noted that several studies found that the association between early ADHD symptoms and later gaming addiction did not remain significant when many covariates were included (e.g., age, child sex, family functioning, academic achievement, pregnancy factors, aggression, and self-esteem; see Table 2 for details).

### Reciprocal associations

A total of ten studies investigated reciprocal associations. Although these studies are included in the results section above, we mention them again here, because they used a design which provided information about the directionality of the associations. A summary of these studies showed that when controlling for baseline levels, three studies found support for bi-directional associations between digital media use and ADHD symptoms [44, 54, 61], four studies found unidirectional associations between digital media use and later ADHD symptoms [42, 46, 47, 56], two studies found unidirectional associations between ADHD symptoms and later digital media use [40, 62], and one study did not find any significant associations [59].

### Covariates, mediators and moderators

#### Effects of sex and age

As most studies included in this review used sex as a covariate rather than as a moderator, we still do not know to what extent the association between digital media use and ADHD symptom levels is stronger for boys than for girls or vice versa. However, Baumgartner et al. [47] showed that the relation between media multitasking and later ADHD symptom levels was twice as large for girls as for boys, and Rydell and Brocki [50] found that symptoms of inattention predicted violent media use 1 year later for boys only. Finally, one study [56] failed to find a moderating effect of sex.

Regarding effects of age, only one study [56] conducted a proper moderation analysis, and it failed to find a moderating effect of age in a sample of children aged 2–6 years when examining screen time in relation to later ADHD symptoms and vice versa. In addition, five studies examined to what extent the association was significant in one age group and not in another. Allen et al. [52] found that screen time was only related to later ADHD symptom levels at age 10 and not age 6. In addition, Baumgartner et al. [47] found an effect of multitasking on later inattention only for children 11–13 years of age and not for teenagers 13–15 years. Hygen et al. [42] found that gaming addiction at age 10 was related to ADHD symptom levels at both age 12 and 14. Liu et al. [49] found that screen time at both 6 months and 2.5 years was related to ADHD symptom levels at age 4. Finally, Stenseng et al. [40] found that ADHD symptom levels at age 6 were associated with screen time at age 10, but that ADHD symptom levels at age 10 were not significantly associated with screen time at age 12. In conclusion, the results are very mixed, and there is little evidence to support the claim that effects are generally stronger for a specific age.

#### Additional variables

As shown in Table 2, the studies included in this review included a large range of different covariates in addition to age and sex. For example, Allen et al. [52] showed no moderating effect of parental income, pubertal status and upbringing, and Hetherington et al. [48] found no moderating effect of either hostile/ineffective parenting or positive parenting. Both these two studies examined the association between screen time for video games/TV and later ADHD symptoms. However, Peeters et al. [66] found that the effect of attention problems in relation to later symptoms of IGD was stronger for adolescents who had fewer close relationships and who were less satisfied with life.

Regarding mediators and covariates, there are studies showing that only the effects of school performance and peer problems [63] or sleep problems [53] remained significant when investigating the association between digital media use and later ADHD symptoms. Finally, Jeong et al. [65] showed support for a mediating effect of low self-esteem for both boys and girls and aggression for girls when investigating the relation between ADHD symptoms and later symptoms of IGD. None of the studies investigated underlying neuropsychological deficits (e.g., executive functioning, emotion regulation, or reward sensitivity) as possible mediators in the association between digital media and ADHD symptom levels.

It should also be noted that other studies included in the present review included a very large range of control variables. These include family climate, intelligence, child temperament, emotion regulation, passive smoking, outdoor activities, school performance, self-esteem, pregnancy factors, executive functioning, physical activity, and social skills (see Table 2 for details). The relation between digital media use and ADHD symptom levels generally disappeared when including a large range of covariates, which indicates that at least some of these factors are of importance. However, because these control variables were entered in the same step, it is not possible to know how each variable affected the relation between ADHD and digital media use.

#### Effects of rater

As mentioned in the method section, all studies but three used ratings to assess ADHD symptom levels and/or digital media use. A majority of the studies also used the same rater for both the predictor and the outcome measure, with 11 studies using only self-ratings and 8 studies using only parent ratings. Associations can be over-estimated if the same rater is used to assess both the predictor and the outcome, and the validity of youth self-reports of ADHD symptom levels can also be questioned. We therefore conducted additional sensitivity analyses to determine whether results differed depending on what type of rater that was used. The results showed that there were no systematic differences between studies reporting significant and non-significant associations with regard to whether the study used parent ratings, self-ratings, or a combination of raters.”

## Discussion

The aim of the present review was to summarize findings from longitudinal studies published during the past 10 years that have addressed the association between digital media use and ADHD symptoms. We found 28 studies meeting our inclusion criteria: 10 addressed reciprocal relations between digital media use and ADHD symptoms, 9 addressed only the association between digital media use and later ADHD symptoms, and 9 addressed only the association between ADHD symptoms and later digital media. The present results showed that a majority (74%) of the studies found significant associations between digital media and later ADHD symptom levels. Effects sizes were often relatively small (e.g., correlation coefficients < 0.30). However, as discussed further below, even small associations may be of importance when controlling for baseline levels in longitudinal studies. In addition, the present review found at least partial support for reciprocal associations, suggesting that ADHD symptoms are related to an increased risk of developing problematic use of digital media, which in turn can exaggerate both their symptom levels and daily life problems due to both the directs effects (e.g., multitasking, quick rewards) and the indirect effects (e.g., negative effects on sleep and social relations) of digital media. In the discussion below, we point to some important limitations of previous research that should be addressed in future studies aimed at better understanding the complex relations between digital media use and ADHD symptoms.

### Factors to take into consideration when interpreting the results

Generally, both the associations between digital media use and later ADHD symptoms and the reverse associations were relatively small. This could be taken to indicate that digital media use and ADHD are not strongly linked. However, there are several important aspects to take into consideration when interpreting the results of the present review. First, it should be noted that the size of the associations depended on what type of digital media that was in focus. In the present review, eight of the nine studies investigating digital media addiction found some significant associations, whereas effects were less consistently found when examining screen time. This could indicate that it is primarily when digital media use has negative consequences for daily life functioning that it is associated with ADHD symptom levels. However, we believe that parents need to monitor their child’s digital media use carefully as early prevention is important and parents play a vital role in teaching children how to use digital media in a balanced way [69, 70].

Second, it has been argued [71] that when controlling for baseline levels in longitudinal studies, even small associations should be considered meaningful when there is high stability in the outcome variable. In the studies included in this review, correlations between ADHD symptom levels at different time-points were high and substantially higher compared to correlations between digital media across time-points. Thus, when investigating relations between digital media and later ADHD symptom levels and controlling for baseline ADHD symptom levels, there is relatively little variance left to explain.

A third issue relates to differential associations. As will be discussed in more detail below, few of the examined studies investigated the role of moderators. Thus, the relatively small associations referred to above apply to the average associations for the entire sample. However, there are likely subgroups of children with ADHD who are at relatively high risk of developing digital media addiction and identifying this “at-risk” group should be of high priority for future research.

A fourth important issue to consider when assessing the size of effects is the time lag between baseline and follow-up. Some digital media effects can have immediate consequences (e.g., cyberbullying or sexual harassment), whereas other effects (e.g., multitasking, playing games with high arousal) take considerably longer to manifest themselves. Many studies included in the present review covered a time span of 1–2 years, which may seem sensible. However, it is possible that some associations might have been missed, because the time span was too short or that the main part of the effect occurred shortly after use began and then remained stable.

A fifth issue concerns the use of more advanced statistical methods that can reveal to what extent associations can only be found for between-subject effects (i.e., how average media use across many children are related to the average ADHD symptom levels of the same children) or whether there is also evidence of within-subject effects (i.e., child X’s media use is related to child X’s ADHD symptoms across time). Only four studies included in the present review investigated both these two types of associations, and two of these studies also found support for within-subject associations. Another important statistical issue relates to control for baseline levels in the outcome measure. In the summary of the results presented in Fig. 2, we demonstrated that results varied substantially based on what type of analyses that were conducted. When interpreting the results of the present review, it should therefore be important to take into consideration that only a small minority of the studies conducted within-subject analyses, which is the type of analysis that provides the strongest support for causal associations.

Even when conducting within-subject analyses, we need to be careful to not interpret the results of the longitudinal studies included in this review as necessarily providing evidence of causal relations. It is possible that associations are driven by other variables, not included in the studies, which are related to both digital media and ADHD symptom levels. Although the study by McNamee et al. [45] showed that associations remained significant when including many different covariates and running several different types of sensitivity analyses, this is an issue that needs to be further addressed in future research.

Finally, it should be noted that only two studies included in the present review investigated non-linear relations. Although, both these studies [40, 45] failed to find evidence of non-linear relations between digital media use and later ADHD symptoms, there are strong reasons to believe that a certain level of digital media use is needed to produce negative effects. By focusing only on linear associations, effects might be underestimated. A previous meta-analysis [72] even found that moderate use of digital media (i.e., less than 2 h/day) was related to lower levels of mental health problems compared to no digital media use at all, while the reverse was true for more excessive use. As using digital media has become the norm in today’s society, not using digital media at all, or not being allowed to use certain games or mobile applications commonly used by the child’s peers, might even have negative effects on peer relations and mental health.

### Direction of associations

One of the great advantages of longitudinal studies is that they can inform us about the direction of the associations. In analyses controlling for baseline levels, the present review found support for a significant relation between digital media and later ADHD symptoms in 12 of 19 (63%) studies and support for the reverse relation in 10 of 19 (53%) studies. However, the best design for studies investigating the direction of associations is of course to look at bi-directional relations (i.e., how digital media and ADHD symptoms mutually influence each other over time). In the ten studies using this design, bi-directional relations were found in only 3 of 10 (30%) studies. In the remaining studies, four studies found support for a relation between digital media use and later ADHD symptoms (i.e., media effects rather than selection effects), two studies found support for the reverse relation, and the last study did not find any significant relations. When interpreting these results, there are some methodological issues that need to be considered. As discussed above, measures of ADHD symptoms are more highly correlated across time compared to digital media use, making it more difficult to find an association between digital media use and later ADHD symptoms compared to the reverse effect [4]. As discussed briefly above, the issue of the time lag between baseline and follow-up is also important to consider, especially in studies investigating bi-directional associations. If selection effects (i.e., ADHD in relation to later digital media) take longer or shorter to manifest themselves compared to media effects (i.e., digital media in relation to later ADHD symptoms), a fixed time lag cannot take this into consideration, possibly resulting in an underestimation of some associations. Effects of ADHD in relation to later digital media use most likely take some time to manifest themselves. With regard to effects of digital media use on later ADHD symptoms, it has been argued that some direct effects, such as those of violent media, are apparent quickly [4], whereas effects of, for example, multitasking [47] take longer. In conclusion, there seems to be relatively strong support for an association between digital media use and later ADHD symptoms as well as vice versa. However, more longitudinal studies examining reciprocal associations are needed, as we were only able to locate ten such studies and these studies showed mixed findings.

### Moderators, mediators and covariates

As concerns sex differences, the present study did not focus on main differences with regard to digital media use or ADHD, but rather on to what extent the associations between digital media use and later ADHD symptoms or vice versa are stronger for one sex compared to the other. Unfortunately, the present review does not shed much light on this issue due to the limited number of studies using sex as a moderator. However, one study found support for a stronger association between violent media and ADHD symptoms among boys [50], and another study found some support for a stronger association between media multitasking and ADHD symptoms among girls [47]. These findings may seem contradictory at first, but they are most likely related to the fact that Baumgarten et al. [47] investigated media multitasking (i.e., an activity more common among girls), whereas Rydell and Brocki [50] investigated screen time for violent media (i.e., an activity more common among boys), and interaction effects are more easily detected in samples with a large range in the variables of interests. However, we would like to emphasize that the significant interaction effects of sex were small and that another study included in the review [56] failed to find a significant moderating effect of sex, suggesting that sex is not a very important moderator in the association between digital media use and ADHD.

As concerns age, the studies included in the present review included children from 18 months to 17 years. Significant associations were found in some studies of younger children [48, 49], but not in other studies looking at children of a similar age [58]. The results were mixed in studies of older children and adolescents as well. Because adolescence is a period of substantial restructuring of the brain, it has been argued to be an especially sensitive period, also regarding the influences of digital media use [73]. Early adolescence is also the period during which many children begin using digital media to a much greater extent than previously, with some official statistics [e.g., 74] showing that “age 13 is the new 16” (i.e., that digital media use now peaks already at age 13 compared to the previous peak at 16 years). Some support for this notion was found in the present review, as Baumgarten et al. [47] found associations between digital multitasking and ADHD symptoms for younger but not older adolescents. In addition, some have argued that effects should instead be the greatest in preschool children, owing to the rapid cognitive and socioemotional development that takes place during this period in life, and research has shown that early screen time does have effects on cognition [e.g., 75], externalizing behaviors [76] and attentional networks in the brain [77]. Interestingly, previous research has found associations even when infants are only exposed to a TV in the background [e.g., 78] or when parents are disrupted by their digital media devices when interacting with their preschool child [e.g., 79].

Regarding additional moderators and mediators, Barlett et al. [53] showed that digital media use was associated with sleep problems, which in turn was associated with increased ADHD symptom levels. Their results are in line with previous studies demonstrating a link between sleep and both digital media use [32] and ADHD [80]. Somewhat surprisingly, we found no longitudinal studies investigating the mediating role of neuropsychological functions, such as executive functions (i.e., working memory, inhibitory control, and planning), delay aversion and emotion regulation, given that these functions have been linked to digital media use [81–83], other types of additions such as alcohol/drug addiction or gambling [84, 85], and ADHD [86]. Given that ADHD is a highly heterogeneous disorder, it is important that future research both identify subgroups with the highest risk of developing digital media addiction and better adapt treatment to meet the needs of individual patients.

### Directions for future research

Although we believe that the present review provides important information, there are some important aspects that require further investigation. First, associations often became non-significant when different covariates were included, which indicates that at least some of these factors are of importance for the association between digital media use and ADHD symptoms. However, rather than including a large range of variables simultaneously, their role as potential mediators or moderators in the relation between digital media use and ADHD symptoms needs to be investigated. We recommend that multiple mediation/moderation models, which promote a better understanding of the complex associations between digital media use and ADHD, be used. Second, we need to move away from investigating only screen time or symptoms of digital media addiction and focus on what type of media use is most problematic and what media activities might even promote positive development. Some of the studies included in the present review did not aim to specifically investigate associations between digital media and ADHD symptom levels, and this was just briefly assessed within a larger study with a much broader aim. To obtain more detailed information, future studies also need to include more precise measurements. As emphasized previously [87], using several types of measures in the same study should also be considered important, as different measures have different strengths and weaknesses. We recommend that future research uses new technological advancements to obtain more detailed information on what type of digital media content children encounter and how this contributes to later mental health outcomes. One study included in the present review [57] provided some insights into this issue by showing that the types of media activities with the highest odds ratios in relation to ADHD symptom levels were video chatting, playing games with yourself, and online shopping/browsing. The lowest odds ratio was found for sending text messages.

Third, we need more longitudinal studies investigating effects of social media in relation to ADHD symptom levels. Although a few studies included in the present review examined Internet addiction (i.e., a combined measure of all types of digital media activities), only two of them [45, 46] focused specifically on social media.

Fourth, we need to use measures other than ratings and use different raters for the predictor and the outcome. Only three reports included in the present review [40–42] used interviews to assess digital media addiction and/or ADHD symptom levels, and this should clearly be considered an important strength of these studies. It should also be noted that the quality criteria indicated that only eight studies used different raters for the predictor and the outcome, which means that associations might have been over-estimated due to rater biases.

Fifth, it is also important to emphasize that longitudinal studies such as those included in the current review need to be complemented with studies using experimental designs to obtain more detailed information about factors that trigger and maintain a problematic use of digital media in children with ADHD. Finally, none of the studies in the present review included clinically referred samples of children diagnosed with ADHD, and this should be considered an important avenue for future longitudinal research.

### Clinical relevance and implications

The results of the present review support the “Differential susceptibility to media effects model” [24], which states that some individuals are more vulnerable to developing problematic use of digital media. Some support was also found for the “Reciprocal Spirals Model” [10], which states that digital media and ADHD have reciprocal effects across time, meaning that using digital media when you have ADHD can result in increased symptom levels over time. As described in the present introduction, it has been hypothesized that excessive digital media use can also cause a range of displacement effects, such as low levels of physical activity, sleep problems, and poor eating habits. Because these lifestyle factors are associated with ADHD, we believe that it is important for clinicians to discuss digital media habits as one potential factor contributing to both increased ADHD symptom levels and exacerbated daily life problems. It should also be noted that once unhealthy life habits have been formed, they are very difficult to change. Thus, parents of children with ADHD need to be aware that their children are at higher risk of being attracted to digital media and that there are characteristics found in many types of digital media (both games and social media) that are more rewarding for children with ADHD, making these children more prone to developing addiction problems. In addition, extensive use of digital media can exacerbate ADHD symptom levels as well as comorbid problems, both directly due to the characteristics of the media (e.g., violent content, fast pace, multitasking, and quick rewards) and indirectly through the negative effects digital media use has on, for example, academic achievement and social relations. Recommendations for digital media use for children have been developed [e.g., 88] and they often include no screen time at all for children under the age of 2, 1–2 h of only high-quality media between age 2–5, and consistent limits even for older children, the goal being to ensure that digital media use does not have negative effects. Importantly, previous research [e.g., 89, 90] has shown that, in many countries, the average screen time for children greatly exceeds these limits, with averages continuously increasing and families with low SES being less likely to follow recommendations. Thus, it is necessary to work actively with parents, schools, and the healthcare sector to promote greater awareness and support children at-risk of developing digital media addiction. During the past few years, politicians and authorities in several countries have also begun discussing the need for new legislation that will force the tech industry to exclude game characteristics that are particularly addictive or detrimental to mental health. These characteristics include infinite scrolling, autoplay, rewarding users for merely using their services, and photo filters that encourage unhealthy beauty ideals [e.g., 91, 92]. In-game purchasing of loot-boxes is another feature that has been shown to have severe negative consequences for children (e.g., stealing from parents or putting oneself in debt), and the Gambling Health Alliance in the UK [93] has therefore suggested that loot-boxes be banned in games for children. Regardless of whether legislation or mutual discussions with the tech industry are the best way forward, we welcome both more research on and political discussions of how to reduce the harmful effects of digital media use on children.

Finally, we like to acknowledge that the conclusions from this review could open up for speculations of a future differential diagnosis such as “digital media induced ADHD” and rightfully raise concerns of potential consequences thereof (e.g., stigma, validity of the ADHD diagnosis, and right to treatment). We would therefore like to emphasize the need for future studies to investigate such issues with more complex methodology, investigating the impact and role of other explanatory factors (moderating and mediating effects). To what extent digital media can cause an ADHD diagnosis is not what this review has investigated as all included studies investigated ADHD symptom levels (i.e., hyperactivity, impulsivity, and inattention) without taking the other diagnostic criteria for ADHD into account.

## Conclusions

The present review shows that digital media use is related to later ADHD as well as vice versa. Thus, these associations are best characterized as reciprocal, in that digital media and ADHD symptom levels affect each other in a complex relation over time. Even though these associations are sometimes small, they should be regarded as important, because they are found rather consistently across studies. Due to the fact that ADHD symptom levels are highly correlated across time, there is also little variance left to explain when investigating digital media in relation to later ADHD symptom levels and controlling for ADHD symptoms at baseline. The present review further shows that associations with ADHD appear stronger for longitudinal studies investigating problematic use of digital media compared to those focusing on screen time. Associations do not appear to be strongly related to either the age or sex of the child. However, the fact that some relations did not remain significant when including covariates could be taken to indicate that there are certain subgroups of children with ADHD symptoms that are more vulnerable to the effects of digital media. Conducting both moderation and mediation analyses should be an important avenue for future longitudinal research if we are to identify subgroups as well as underlying factors that can better explain the link between digital media use and ADHD symptom levels.

## Data Availability

No applicable.
